# The role of visual cortex acetylcholine in learning to discriminate temporally modulated visual stimuli

**DOI:** 10.3389/fnbeh.2013.00016

**Published:** 2013-03-20

**Authors:** V. H. Minces, A. S. Alexander, M. Datlow, S. I. Alfonso, A. A. Chiba

**Affiliations:** ^1^Temporal Dynamics of Learning Center, University of CaliforniaSan Diego, CA, USA; ^2^Department of Cognitive Science, University of CaliforniaSan Diego, CA, USA; ^3^Program in Neuroscience, University of CaliforniaSan Diego, CA, USA

**Keywords:** acetylcholine, timing and time perception, plasticity and learning, visual cortex, 192 IgG-saporin

## Abstract

Cholinergic neurons in the basal forebrain innervate discrete regions of the cortical mantle, bestowing the cholinergic system with the potential to dynamically modulate sub-regions of the cortex according to behavioral demands. Cortical cholinergic activity has been shown to facilitate learning and modulate attention. Experiments addressing these issues have primarily focused on widespread cholinergic depletions, extending to areas involved in general cognitive processes and sleep cycle regulation, making a definitive interpretation of the behavioral role of cholinergic projections difficult. Furthermore, a review of the electrophysiological literature suggests that cholinergic modulation is particularly important in representing the fine temporal details of stimuli, an issue rarely addressed in behavioral experimentation. The goal of this work is to understand the role of cholinergic projections, specific to the sensory cortices, in learning to discriminate fine differences in the temporal structure of stimuli. A novel visual Go/No-Go task was developed to assess the ability of rats to learn to discriminate fine differences in the temporal structure of visual stimuli (lights flashing at various frequencies). The cholinergic contribution to this task was examined by selective reduction of acetylcholine projections to visual cortex (VCx) (using 192 IgG-saporin), either before or after discrimination training. We find that in the face of compromised cholinergic input to the VCx, the rats' ability to learn to perform fine discriminations is impaired, whereas their ability to perform previously learned discriminations remains unaffected. These results suggest that acetylcholine serves the role of facilitating plastic changes in the sensory cortices that are necessary for an animal to refine its sensitivity to the temporal characteristics of relevant stimuli.

## Introduction

In order to negotiate the demands of the environment, we differentially allocate our resources according to the contingencies that confront us, sometimes increasing the processing capabilities of a specific modality or modulating processing of certain fields within a sensory region. This increased capability can be manifested with regard to static characteristics of the stimuli, such as orientation or pitch, but also in relation to dynamic characteristics, such as amplitude modulations. Converging evidence indicates that the neuromodulator acetylcholine is a likely contributor to these processes (Metherate et al., 2005).

The basal forebrain cholinergic system contains a continuum of projection neurons that provides the major source of cholinergic input to nearly all neocortical and various sub-cortical regions. Several studies indicate that these projections are roughly organized in a topographical manner (for a detailed review see Zaborszky et al., 1999). Such an organization suggests that this system has the capacity to differentially modulate the activity of cortical regions according to behavioral demands.

A phenomenon closely associated with cholinergic activity is that of brain activation, characteristic of states of high arousal and REM sleep (Steriade and McCarley, 2005). A detailed review of the literature indicates that the most consistent effect of brain activation on sensory responses is augmenting the capacity of sensory neurons to represent time modulated stimuli (Castro-Alamancos, 2004b). An augmentation of the temporal resolution of neuronal responses is also found with specific cholinergic manipulations either *in vivo* or in slices (Gil et al., 1997; Castro-Alamancos, 2004a,b). This indicates that the cholinergic system may play a role in increasing the capacity of a specific cortical region to process time modulated signals.

A variety of experiments examining selective lesions in different regions of the basal forebrain or electrical stimulation of the basal forebrain also provide evidence of at least a roughly topographic distribution of cortical projections. For example, more posterior lesions of the basal forebrain produce greater depletions of acetylcholine in the auditory cortex (ACx) than in the visual cortex (VCx), whereas the converse is true for lesions that are more anterior (Wenk et al., 1980). In accordance with this, electrical stimulation in posterior zones (but not anterior zones) produces EEG activation in auditory areas that is abolished by iontophoretic application of atropine (Metherate et al., 1992). Stimulation of different regions of the basal forebrain also produces differential acetylcholine release in motor or visual cortices (Jiménez-Capdeville et al., 1997). Additionally, evidence exists that cortical sensory regions can be differentially modulated through prefrontal cortex (PFC) projections to the basal forebrain that influence subsequent output to sensory cortices (Golmayo et al., 2003).

Given evidence implicating basal forebrain cholinergic neurons in cortical activation and modulation, numerous behavioral studies on the role of acetylcholine on sensory and motor processing have been done. The majority of these studies focus on rather extended cholinergic depletions (Fine et al., 1997; Linster et al., 2001; Conner et al., 2003; Dotigny et al., 2008) that not only affect the specific modality tested but also higher order cognitive functions (Baxter and Chiba, 1999) as well as sleep cycle regulation (Platt and Riedel, 2011). These studies have found sensory and motor learning impairments in cholinergic depleted animals (when lesions were performed before learning), but no immediate effect on sensory processing or motor activity. Another study using cholinergic lesions restricted to the ACx revealed an auditory sequence discrimination learning deficit in the face of preserved sound discrimination (Kudoh et al., 2004). Despite the wide array of functions examined, none of these lesion studies has utilized temporally modulated stimuli.

The goal of this study is, therefore, to understand the influence of cholinergic activity specific to a sensory area (in this case the VCx) on the ability of an animal to learn to discriminate temporally modulated signals. A second goal is to examine the necessity of VCx acetylcholine in supporting the ability to discriminate these stimuli after they are learned.

In order to induce a focal cholinergic depletion, 192 IgG-saporin was delivered directly into the VCx of experimental rats. Through retrograde transport, this immunotoxin selectively eliminates cholinergic neurons projecting to the target area (McGaughy and Sarter, 7142); while leaving cortical neurons and other myelinated fibers intact (Bucci et al., 1998). In order to evaluate post-operative learning and discriminative capacity, we use a go/no-go discrimination task across a continuum of temporally modulated visual test stimuli, thereby deriving a psychometric curve. This method ensures that if there is a discrimination impairment, the range in which it is observable will be revealed.

## Methods

### Experimental subjects

All procedures and animal care adhered strictly to AAALAC and institutional guidelines for experimental animal health, safety, and comfort. For all experiments we used male, Long-Evans rats (Charles River, Raleigh NC). Rats were maintained in a temperature-controlled room with a 12 h. light/dark cycle (lights on 08:00–20:00). Water was available *ad libitum* and food was controlled, such that weight was maintained at rats' original *ad libitum* weight.

A total of 28 rats were used across two experiments. In the *performance experiment* six rats were assigned to the control group and nine to the depletion group. In the *learning experiment* six rats were assigned to the control group and seven to the depletion group. All studies were performed in an experimenter blind (to lesion condition) manner.

### Surgery

Each rat was anesthetized with isoflurane anesthesia prior to being positioned in a Kopf stereotaxic apparatus. An incision was made along the midline of the rat's head, and the underlying periosteal fascia was swabbed to the side. To target the VCx, we obtained midline measurements at the caudal ridge of the skull; we then measured the A/P coordinates from the caudal ridge at ±2.5 mm lateral to midline and at ±3.0 mm lateral to midline. Subsequently, we measured 1 mm anterior to the ridge at ±2.5 mm lateral to midline and drilled two small holes through the skull (with a No. 1 drill bit) at that location. We then measured 2 mm anterior to the ridge at ±3.0 mm lateral to midline and drilled two additional holes at that location. A small slit was made in the underlying dura at each location, to allow needle penetration.

Selective depletion of VCx acetylcholine was achieved using intracortical infusions of the immunotoxin, 192-IgG-saporin (SAP; Advanced Targeting Systems, San Diego, CA), based on established techniques (Holley et al., 1994; Bucci et al., 1998; McGaughy and Sarter, 7142; Conner et al., 2010). Conner and Chiba jointly piloted the dilution and demonstrated the efficacy of this lot of 192-IgG-saporin. Published photomicrographs using comparable techniques and dilutions to those used in the current study are available in Conner et al. (2010). 192-IgG-saporin was diluted to 0.075 mg/ml in artificial cerebrospinal fluid (ACSF) and delivered using a Hamilton syringe (custom model 701SN, 10 μl, Hamilton Company, Reno NV) with a 30 gauge permanent needle and a 2/3 custom bevel. Immunotoxin injections, or equal volume injections of ACSF vehicle, were made bilaterally, with the head level between lambda and bregma. At each injection site, 0.2 μl was delivered at a rate of 0.05 μ l/min. The needle remained at the injection site for 30 s before and 3 min after each injection. Following the completion of all injections, the skull was cleaned and the area containing the drill holes covered with gel foam. Subsequently, the wound was sutured and application of a topical antibiotic containing lidocaine was applied to the wound area. Each rat was monitored during recovery from anesthesia. Surgeries of lesions and controls were interspersed so as to allow equal recovery time, controlling for possible detrimental effects of the anesthesia. Rats were left to recover between 14 and 20 days prior to commencing the behavioral tasks.

### Behavioral methods

**Figure 1:**
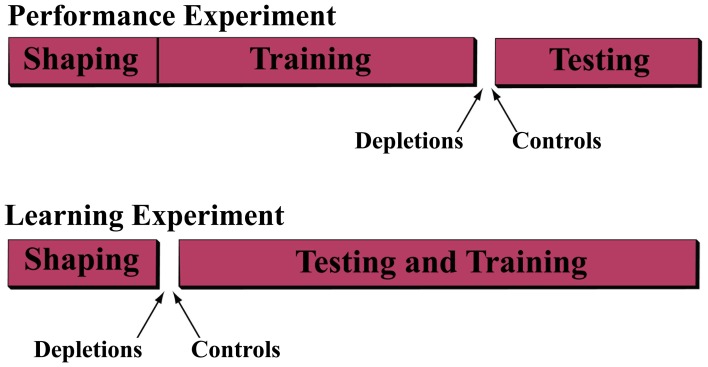
**Experimental scheme.** In order to understand the role of acetylcholine in the ability to discriminate and to learn to discriminate temporally modulated stimuli, we performed two experiments. In the first (*performance experiment*) rats were trained on the *discrimination task* for 25 sessions, after which they were separated into either the cholinergic depletion (192 IgG-Saporin) or control (ACSF vehicle) group and re-assigned to the task for 7 sessions of testing. In the second experiment (*learning experiment*) the rats were assigned to the depletion or control group prior to administration of the *discrimination task* for 25 sessions.

We performed two operant learning experiments; see Experimental Scheme (Figure 1).

In the *performance experiment*, we trained the rats on the *discrimination task*, described below, until they reached asymptotic behavior (the psychometric curves become stable), after which we assigned them to one of two groups. In the *depletion group* we depleted cholinergic input to the VCx by infusion of 192-IgG-saporin and in *the control group* we infused ACSF vehicle. After a recovery period, we re-tested the animals on the task.

In the *learning experiment*, we performed the lesions prior to behavioral training and analyzed the rats' ability to acquire the discrimination.

#### Behavioral apparatus

Experiments were carried out in four Coulbourn Chambers (Coulbourn Instruments, Whitehall, PA) each with dimensions 22.9 × 20.3 × 20.3 cm, aluminum walls, clear acrylic sides and ceiling, and a grid floor (0.48 cm stainless steel rods spaced 1.9 cm apart). A dimly illuminated food cup was recessed in the center of one wall. Visible through the Plexiglas ceiling was a circular array of five, 10 mm ultra-high brightness white LED lights [intensity: 28,500 mcd (typical), viewing angle: 10°], filtered through a paper shade. The resulting light served as the light source of the visual stimulus. The conditioned stimuli consisted of flashes of the described light ring, resulting in an ambient light flash emanating from the ceiling (the area to which the rat reared). The onset of the lights illuminated the entire chamber with the area beneath the ceiling measuring 15.8 candela per square meter. If the rat did not demonstrate the classic Pavlovian response to the light, the light was still visible at 0.35 candelas per square meter off the sidewalls of the chamber and 0.43 at the floor, as compared to 0.01 candelas per square meter with the lights off. Each experimental chamber was enclosed in a sound-resistant shell, and equipped with a ventilation fan that provided masking noise. Webcams were mounted such that the rat could be visually monitored and recorded during each behavioral session. Stimulus presentation, food delivery, and data collection (lever presses) were performed through a National Instruments (National Instruments, Austin, TX) interface, controlled by software programmed in Matlab (by first author, Minces).

#### Behavioral shaping

Four-month old rats were fed *ad libitum* and handled for 2 weeks. Following this adaptation period they were food deprived until they reached 80% of their *ad libitum* weight (approximately 2 additional weeks with continued handling), at which point behavioral shaping began. Following initial shaping, rats were allowed to slowly gain weight until they were maintained at their original *ad libitum* weight.

Shaping sessions were 30 min long. Rats were moved into each successive shaping stage as long as they maintained motivation (worked for more than 30 rewards) or met the criteria specified for each stage. If a rat did not meet the criteria, he was moved back into the previous stage.

Stage 1—Rats were placed in the training boxes with only the food port present. Sugar pellets were placed in the food port and the rats were left to eat at will. Once they were comfortable enough to eat in a sustained manner from the wells (about two sessions) they were moved into the next stage.

Stage 2—Rats were put in the box with the food port empty. A pellet would drop every minute, and the rats were visually monitored to determine whether they approached the food port when the pellets dropped.

Stage 3—At this point the lever was introduced and the rats were trained to press the lever for food. In order to facilitate lever pressing, a few pellets were put on top of the lever for the first few trials. Once the rats pressed the lever more than 30 times, in two consecutive training sessions, they advanced to the next stage.

Stage 4—Rats were first trained to press the lever to a solid (not flashing) light. This was done in the following manner: solid lights were presented for 20 s followed with 20 s of darkness; the rats were only rewarded if they pressed the lever during the light period. If they pressed the lever during darkness, the time until the subsequent light was delayed for 1 s. In this manner the light was never presented while the rat exhibited extra lever presses, thus prohibiting incidental reinforcement of the rat pressing the lever during darkness.

Stage 5—The light was set to turn off when the lever was pressed (and the pellet delivered).

Stage 6—The light period was set to 10 s and the darkness to 20 s.

Stage 7—The presentation of the light was randomized in such a way that it was only presented in 50% of the instances. In the other 50% the box remained dark. Rats were considered to have completed the shaping procedure if they pressed the lever in response to more than half of the light presentations, and also pressed the lever during darkness less than half the number of times that they pressed during light presentation.

Total shaping time was 10–15 sessions, after which the rats were brought back to their pre-adjusted weight. At this weight, rats remained appropriately motivated (working for a full hour without excessive impulsivity) throughout the rest of the experiment. Before the *discrimination task* was initiated, rats were exposed to a 60-min session similar to shaping stage 7, but using a 12 Hz flashing light as opposed to a solid light.

#### Discrimination task

Rats were placed in the above described chambers and presented with the following set of stimuli: a CS+ (a stimulus that was reinforced with delivery of a sucrose pellet) consisting of a 12 Hz flashing light that was presented 25% of the times, a CS− at 2.12 Hz that was presented 20% of the times, and a set of 20 intermediate frequencies ranging from 3.78 to 11.32 Hz, arranged according to a chromatic scale such that the frequency ratio between two adjacent stimuli was held constant and that a gap of 12 frequencies corresponded to a ratio of 2. The intermediate frequencies (in Hz) used were as follows:

3.78, 4.00, 4.24, 4.45, 4.76, 5.05, 5.35, 5.66, 6.00, 6.36, 6.73, 7.14, 7.56, 8.00, 8.49, 8.99, 9.52, 10.09, 10.69, 11.33.

The intermediate frequencies were presented in a pseudo-random order, in such a way that at the end of each session they were all presented approximately the same number of times. Because the periods of darkness (during the flashes) were of equal duration to the periods of light, the mean intensity of all flashes was constant. We call these intermediate frequency stimuli Test Stimuli, and number them from 1 to 20.

This arrangement allowed for there to be a number of presentations of the CS+ and the CS− such that the rats could learn the contingencies without losing motivation either for lack of sufficient reward or for pressing the lever too many times without a reward. All sessions were 1 h long.

#### Performance experiment

A total of 15 animals participated in this experiment.

Rats in this experiment went directly from shaping into the *discrimination task*. They were run on this task for 25 sessions, after which they were split into a depletion group (*N* = 9) and a control group (*N* = 6), matched according to the mean psychometric area (PSA) of the last 7 days (see quantitative methods). After recovery, they were tested on the same task for 7 days.

#### Learning experiment

A total of 13 rats participated in this experiment. After the shaping procedure rats were randomly assigned to the lesion (*N* = 6) or control (*N* = 7) groups. Surgeries were performed and after recovery, rats were run on the *discrimination task* for 25 days.

#### Total duration

The total number of sessions of shaping and training was between 35 and 40, (depending on the duration of the shaping period), approximating a period of 6 weeks.

### Histology

At the completion of behavioral testing, rats were given a lethal injection of sodium pentobarbital and perfused through the ascending aorta with 400 ml of 0.1 M phosphate buffer at pH 7.4, followed by a solution of 4% paraformaldahyde into an equivalent phosphate buffer solution. The brains were removed from the skulls and stored in a 4% paraformaldehyde-20% sucrose solution for 3 days prior to sectioning. Brains were cut into 50 μm sagittal sections on a freezing microtome to assess the accuracy and extent of the lesions. Sections were processed for acetylcholinesterase (AChE) histochemistry, using acetylthiocholine iodide (Sigma-Aldrich, St. Louis, MO) and following a protocol adapted from (Karnovsky and Roots, 1964) to reveal the presence or absence of cholinergic fibers in the cortex, and to provide an observational approximation of the degree of cholinergic denervation in the acetylcholine depleted rats (see Chiba et al., 1995 for details). AChE stained sections were mounted onto gel-subbed slides, dehydrated in a graded series of alcohols, cleaned and cover-slipped. Based on histochemical staining, an observer blind to the experimental condition independently assessed the approximate extent of depletion in the visual cortices of each brain (0–100%).

### Quantitative methods

#### Overall response

The probabilities of pressing for the CS+, Pr(CS+), or the CS−, Pr(CS−), were estimated as the number of presses to each stimulus divided by the number of presentations. Pr(CS+) and Pr(CS−) were assessed separately from the corresponding values for the test stimuli, because they provide an overall measure of the motivation of the animal, and because these stimuli are presented many times in any single session (more than 20 each), providing a more accurate estimation of each rat's baseline probability of responding.

#### Psychometric function

We calculated the psychometric curves as the estimated probability of responding for each stimulus (presses/presentations), normalized to the probability of responding for the CS+.

$$\text{Ps}(n)=\frac{\text{Pr}(n)}{\text{Pr}(\text{CS}+)}$$

where *n* represents the stimulus number (1–20).

We employ this normalization to control for slight changes in the motivation of individual animals, and because we are interested in how much an animal can discriminate any given stimulus from the CS+. As there was no difference in the amount of pressing for the CS+ between lesion and control animals, this normalization should not introduce a bias for any group.

Because the number of presentations of each test stimulus within a given session is relatively low (about three presentations), the data for each single session is very noisy. In order to overcome this we averaged the psychometric curves of each rat in running blocks of five sessions. When this average is not performed the results are qualitatively similar.

#### Psychometric area

While the psychometric functions provide a lot of information, they do not explicitly inform us of how well the animal is performing. In order to quantify this we define the PSA as the area under the psychometric curve (excluding the CS+) divided by the total number of stimuli presented.

$$\text{PSA}=\frac{\text{Ps}(\text{CS}-)+\sum_{n=1}^{20}\text{Ps}(n)}{21}$$

Thus, defined, the PSA is 1 if the animals press the same amount to all stimuli, and 0 if they press to the CS+ only.

#### Stability of the behavior in the performance experiment

In order to assess when the behavior in this experiment becomes stable we compare the PSA in a given session with the 5th session following it.

### Statistical methods

To assess the statistical significance of the Pr(CS+) between lesions and controls we ran a Kruskal–Wallis test, a non-parametric analysis of variance. When comparing psychometric curves between two groups we ran the Kruskal–Wallis test for each frequency. To compare the PSAs we ran a Kruskal–Wallis test for each session or, on averaged blocks, for each block.

## Results

### Histology

**Figure 2:**
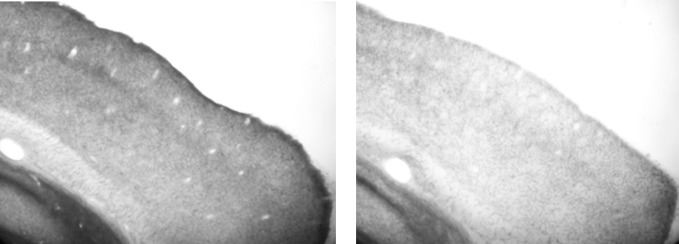
**Representative sagittal sections stained for acetylcholinesterase. Left panel:** visual cortex of ACSF infused rat. **Right panel:** visual cortex of 192 IgG-saporin infused rat, demonstrating lighter staining. Note that both sections were processed as part of the same assay, in the same plate, with the same stop time.

After the experiment was complete we performed histology and the efficacy of the lesions was confirmed by a blind (to lesion group) observer. Figure 2 shows a representative example of the VCx of a control rat and that of a depleted rat. Visual inspection ratings resulted in the average cholinergic depletion being estimated at 70%. Each brain was designated to have a particular percent depletion based on the approximate density of stained fibers in primary VCx (at 20× magnification, on brightfield, using a Nikon E600 Microscope), relative to a “standard” control brain processed in the same histochemistry dish. Equivalent depletions were sufficient to abolish map plasticity in motor cortex (see Conner et al., 2003).

### Performance experiment

#### Pre-lesion

After a few sessions in the discrimination task all animals began to inhibit pressing to frequencies that were most distinct from the CS+.

By session 25 the rat's behavior was stable as revealed by the fact that only sessions one to twelve and session fifteen are statistically different from their shifted counterparts (*p* < 0.05).

#### Effect of cholinergic depletions

**Figure 3:**
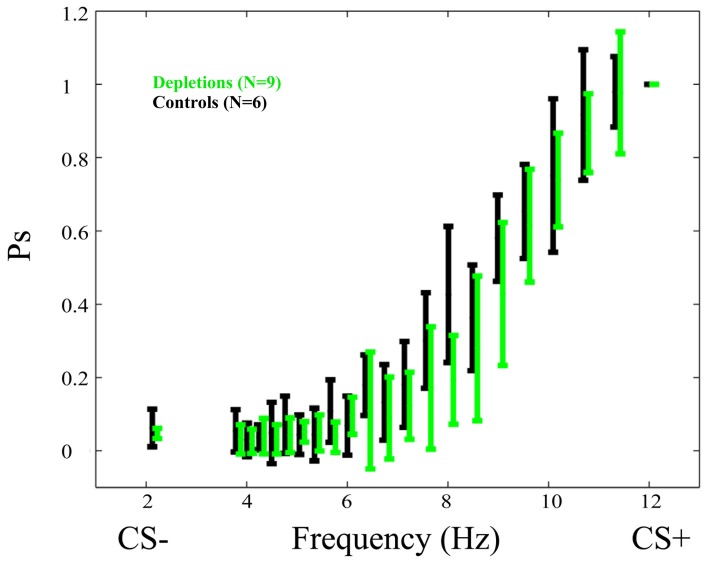
**Psychometric curves in the test phase of the *performance experiment*.** Data for depletion (192 IgG-Saporin) and control (ACSF) groups are depicted in green and black, respectively. In this experiment depletions were performed after learning had taken place. The fact that the psychometric curves do not differ significantly is an indication that cortical acetylcholine has no direct effect on the ability of rats to discriminate temporally modulated visual stimuli (lights flashing at different frequencies). Ps is the probability of responding to a given frequency normalized to the response to the CS+. The Frequency (Hz) represents the frequency at which the stimulus is flashing. Error bars represent the standard deviation.

Psychometric curves in the testing phase (Figure 3) show no difference between lesions and controls. There exists no frequency in which the difference is significant *p* > 0.05.

Further, the post-lesion PSAs, normalized to the pre-lesion values of each rat, do not differ significantly between groups *p* > 0.4.

### Learning experiment

After completion of surgeries (depletions and control) and a corresponding recovery period, the animals were run on the *discrimination task*.

#### Stimulus detection

The rats' motivation to perform the task and general capacity to detect stimuli was not affected by cholinergic depletions, as revealed by the fact that percent pressing to the CS+, Pr(CS+) did not differ significantly among groups in any of the sessions. The mean Pr(CS+) across all sessions is 0.83, revealing that the rats remain motivated to perform the task.

#### Rule learning

Both groups of animals can learn to discriminate stimuli that are sufficiently different equally well, as revealed by the fact that Ps(CS−) was not different between groups in any of the sessions (*p* > 0.05). This emphasizes the rats' intact abilities to learn the rules necessary to make a basic discrimination.

#### Fine discrimination learning

**Figure 4:**
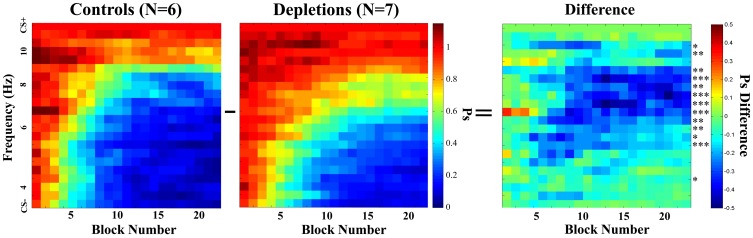
***Learning experiment*, temporal evolution of the psychometric curves.** Comparison of psychometric curves of the control (ACSF vehicle) and the cholinergic depletion (192 IgG-Saporin) groups as they evolve over time (training block number). Each block is a rolling average of 5 sessions. After a few sessions, the animals in both groups begin to inhibit responding to the frequencies that are the most distant from the CS+. Once the psychometric curves become stable, it is evident that the acetylcholine-depleted rats demonstrate poorer discrimination for frequencies that are midway between the CS− and the CS+. This indicates that acetylcholine selective to the visual cortex can facilitate learning to discriminate fine differences in the temporal characteristics of the stimuli. The rightmost graph corresponds to the difference between the two groups; asterisks represent the statistical significance of the differences in the last block, ^*^p < 0.05, ^**^p < 0.025, ^***^p < 0.01.

**Figure 5:**
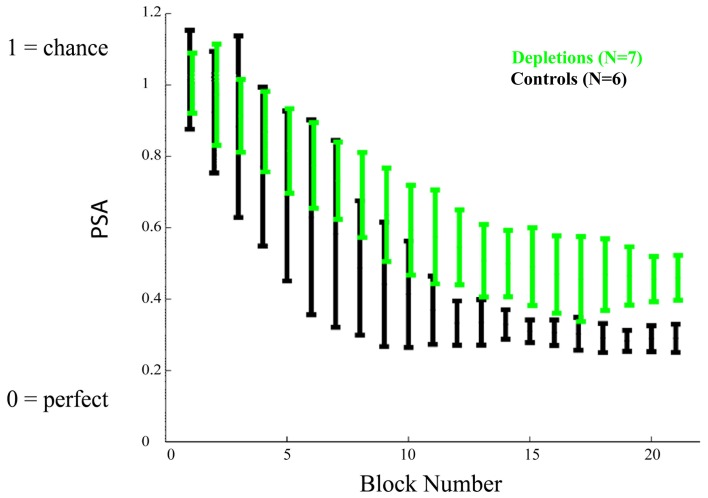
***Learning experiment*, temporal evolution of the psychometric area.** The psychometric areas (PSA) represent the area underneath the psychometric curves (the psychometric curves are depicted in Figure 4). A PSA of 0 corresponds to perfect discrimination (responding to the CS+ only) and a PSA of 1 corresponds to no discrimination (responding equally to all stimuli). Acetylcholine depleted rats demonstrate a significant impairment in performance after block 12, *p* < 0.02. Error bars represent the standard deviation.

As we established that there are not general differences in motivation, target detection, or rule learning, we proceeded to investigate the rats' response to all frequencies. Figure 4 shows the mean Psychometric Curves of depletions and controls for running blocks of five sessions. We analyzed the differences between groups according to the PSAs, which can be seen in Figure 5. The controls perform better than the lesions from block 12 on (*p* < 0.02), demonstrating impairments in learning to make fine discriminations.

## Discussion

The reported results indicate that cholinergic input, restricted to visual cortical areas, is not essential for the correct discrimination of temporally modulated visual stimuli (Figure 3). This is in contrast to the measurable differences observed between groups when the cholinergic depletions are performed prior to learning to discriminate (Figures 4 and 5). The results of our experiments demonstrate that both cholinergic depleted and vehicle control rats can learn the visual discrimination task and that they do so at an equivalent pace, evidenced by the fact that there is no difference in the amount of pressing for the CS+ and for the ratio CS−/CS+. However, after a few training sessions a clear pattern emerges in which the cholinergic depleted animals cannot achieve the same level of discrimination refinement as their intact counterparts (Figures 4 and 5).

These findings are in agreement with previous work demonstrating that cortical acetylcholine plays a role in learning but not in performance *per se* (Conner et al., 2003; Kudoh et al., 2004; Wilson et al., 2004). However, because we test discrimination parametrically across a continuum of task difficulties, our methods are well-suited to detect subtle differences that might have gone undetected in previous studies. Our results suggest that fine differences in the temporal characteristics of the stimuli can, indeed, be resolved without cholinergic input. Conversely, we demonstrate that *learning* to detect subtle differences relies on intact cholinergic input to VCx. In contrast to previous studies (Kudoh et al., 2004), the observed learning impairments are not specific to sequences of different stimuli but are instead expressed toward simple temporally modulated signals. Because we use cholinergic depletions that are specific to the sensory modality that is being tested, we are able to rule out that any observed impairment is caused by the denervation of larger networks, including areas associated with higher cognitive functions or sleep regulation. Our results regarding the preserved ability to process stimuli modulated at these frequencies might seem to contradict electrophysiological evidence, in which it is shown that cholinergic-associated brain activation improves the ability of cells to follow high frequency sensory stimulation (Castro-Alamancos, 2002a, 2004a,b; Castro-Alamancos and Oldford, 2002; Marguet and Harris, 2011). We offer several possible explanations for the observed disagreement.

One possibility is that, even if an activated cortical state is strongly associated with cholinergic activity, acetylcholine may not play an important role in shaping the sensory responses that are observed during the activated state. This is supported by the finding (Goard and Dan, 2009) that the muscarinic antagonist atropine can locally disrupt cortical activation while leaving the reliability of individual neural responses relatively unaffected. Still, this explanation is not entirely satisfactory given that other groups have shown a direct influence of acetylcholine on shaping sensory responses (Roberts et al., 2005; Disney et al., 2007; Kawai et al., 2007).

Despite the fact that it has been shown that applying atropine locally to the cortex can abolish cortical activation (Metherate et al., 1992; Bakin and Weinberger, 1996; Goard and Dan, 2009), studies that chronically abolish cortical acetylcholine have failed to show such an effect (Nilsson et al., 1992; Kaur et al., 2008). The mechanism by which acetylcholine depleted brains can retain or regain activation is currently an open and pressing question in the field. Here, several issues might be considered, including the possibility that acetylcholine is not necessary for activation and that the atropine dosage used in the past was too large, thereby causing non-specific receptor-binding effects. It is also possible that when cortical acetylcholine is chronically abolished other neuromodulatory systems compensate, such as serotonin (Dringenberg and Vanderwolf, 1998). Given that cholinergic depleted rats demonstrate discrimination learning, but not discrimination deficits, it is reasonable to hypothesize that the role of acetylcholine as a modulator of performance can be compensated for but its role as a facilitator of plasticity cannot.

Although in smaller magnitudes, it has been shown that non-visual areas of the cortex can respond to visual stimuli (Wallace et al., 2004), it is therefore possible that non-depleted regions of the brain might be compensating for the loss of function in VCx. If this is the case, it is remarkable that this compensation exists for already acquired discriminations but not for learning. We note that the effect that we observe for learning but not performance is in line with previous works in which depletions were not area-specific (Conner et al., 2003; Wilson et al., 2004).

Previous studies have emphasized the importance of cortical ACh in neuronal responding to spatial patterns (Roberts et al., 2005) and spatial stimulus contrasts (Disney et al., 2007). ACh has also been hypothesized to sharpen auditory receptive fields (Metherate et al., 2005), and cholinergic-associated brain activation has been shown to sharpen the somatosensory receptive fields (Castro-Alamancos, 2002b). In light of this, we might expect that cholinergic depletions could alter the way in which rats respond to patterned stimulation. Because we are interested in the role of VCx acetylcholine in time rather than space modulation, we chose a non-patterned, diffuse light stimulus. However, it might still be useful to investigate the effect of ACh in spatial responses by performing a similar experiment to ours only using static patterns of various spatial frequencies or contrasts, in order to extend our hypotheses to the spatial domain.

Cortical acetylcholine has also been associated with modulation of attention in a variety of behavioral contexts (see Baxter and Chiba, 1999; Sarter et al., 2005). The results of our experiments could be interpreted as relevant to this body of work, in that it is possible that a form of attention facilitating visual learning may be disrupted by cholinergic depletion. Because the task demands of our *performance experiment* and our *learning experiment* are equivalent, it is unlikely that a generalized effect of attention is sufficient to explain our data. Future work parametrically manipulating aspects of attention within the context of our tasks would serve to address this fundamental question.

The cholinergic system has been implicated in attention, neural plasticity, and learning. Except for results in the (anatomically distinct) olfactory system (Linster et al., 2001), previous work has failed to reveal a direct influence of cholinergic activity on sensory perception. The anatomical characteristics of the cholinergic system put it in a favorable position to dynamically modulate the activity across different brain areas (Golmayo et al., 2003). In spite of this, lesion studies have focused on cholinergic depletions of expansive cortical regions and less attention has been given to the influence of acetylcholine specifically on sensory cortical regions. Electrophysiological evidence in anesthetized rats suggests that an important role of cholinergic activity is to regulate the neuronal response toward temporally modulated stimuli (Castro-Alamancos, 2004a,b; Marguet and Harris, 2011). The behavioral effect predicted by these studies is not evident in our findings. Instead, our experiments indicate that the cholinergic system is well-situated to dynamically regulate *learning* of temporal stimuli across different cortical regions.

### Conflict of interest statement

The authors declare that the research was conducted in the absence of any commercial or financial relationships that could be construed as a potential conflict of interest.
